# Evaluation of habitat suitability index models by global sensitivity and uncertainty analyses: a case study for submerged aquatic vegetation

**DOI:** 10.1002/ece3.1520

**Published:** 2015-06-01

**Authors:** Zuzanna Zajac, Bradley Stith, Andrea C Bowling, Catherine A Langtimm, Eric D Swain

**Affiliations:** 1 Department of Agricultural and Biological Engineering, University of FloridaGainesville, Fl, USA; 2 CNB contractor to U. S. Geological Survey, Southeast Ecological Science Center7920 NW 71st., Gainesville, FL, 32653, USA; 3 Department of Fisheries and Wildlife, Michigan State UniversityEast Lansing, MI, 48824, USA; 4 US Geological Survey, Southeast Ecological Science Center7920 NW 71 st., Gainesville, FL, 32653, USA; 5 US Geological Survey, Florida Water Science CenterFt. Lauderdale 7500 SW 36th Street, Davie, FL, 33314, USA

**Keywords:** Everglades, habitat suitability index, sensitivity analysis, Sobol, uncertainty analysis

## Abstract

Habitat suitability index (HSI) models are commonly used to predict habitat quality and species distributions and are used to develop biological surveys, assess reserve and management priorities, and anticipate possible change under different management or climate change scenarios. Important management decisions may be based on model results, often without a clear understanding of the level of uncertainty associated with model outputs. We present an integrated methodology to assess the propagation of uncertainty from both inputs and structure of the HSI models on model outputs (uncertainty analysis: UA) and relative importance of uncertain model inputs and their interactions on the model output uncertainty (global sensitivity analysis: GSA). We illustrate the GSA/UA framework using simulated hydrology input data from a hydrodynamic model representing sea level changes and HSI models for two species of submerged aquatic vegetation (SAV) in southwest Everglades National Park: *Vallisneria americana* (tape grass) and *Halodule wrightii* (shoal grass). We found considerable spatial variation in uncertainty for both species, but distributions of HSI scores still allowed discrimination of sites with good versus poor conditions. Ranking of input parameter sensitivities also varied spatially for both species, with high habitat quality sites showing higher sensitivity to different parameters than low-quality sites. HSI models may be especially useful when species distribution data are unavailable, providing means of exploiting widely available environmental datasets to model past, current, and future habitat conditions. The GSA/UA approach provides a general method for better understanding HSI model dynamics, the spatial and temporal variation in uncertainties, and the parameters that contribute most to model uncertainty. Including an uncertainty and sensitivity analysis in modeling efforts as part of the decision-making framework will result in better-informed, more robust decisions.

## Introduction

Modeling or predicting habitat suitability and species’ distributions is fundamental to research in ecology and conservation. For species for which the presence–absence or abundance data are available, a wide variety of species distribution models (SDM) can be used to find statistical relationships between empirical data on species distribution and environmental data (Elith and Leathwick 2009). However, in many situations distribution data may not be available for species that are poorly studied or difficult to survey. In the absence of distribution data, process-based or mechanistic models are available to model habitat suitability and potential distribution (Dormann et al. 2012). One such process-based approach that has been widely used is “habitat suitability index” (HSI) models. With a lower demand for data compared to other approaches (Burgman et al. 2005), HSI models provide flexible cost-effective decision support tools for natural resource management and ecosystem restoration (Brooks 1997; Burgman et al. 2001). HSI models compute an HSI for flora or fauna species from one or more relevant habitat variables (US Fish and Wildlife Service 1980), and mainly have been used for assessment of reserve designs and potential impacts of management decisions on habitat quality (Burgman et al. 2001). With anticipated increasing climate and land-use change, HSI models may become increasingly important to address management objectives and to utilize the large and rapidly growing body of simulated climate and hydrology datasets.

Despite widespread application of HSI as a management tool (Brooks 1997; Jones-Farrand et al. 2011; Nukazawa et al. 2011), uncertainty analysis is rarely used to evaluate these models (Roloff and Kernoha 1999; Van der Lee et al. 2006). Usually HSIs are calculated deterministically, that is, using best estimates for model inputs, without any consideration of uncertainty of those inputs. However, like most ecological models, HSI model inputs (data, parameters) are uncertain due to process variation (natural spatial and temporal variability) and sampling variation (e.g., inadequate sampling or measurement errors) (Burgman et al. 2001; Van der Lee et al. 2006). The need to quantify uncertainty in HSI models has been highlighted by governmental agencies, such as the U.S. Environmental Protection Agency and the European Commission (Saltelli and Annoni 2010), and numerous researchers (Bender et al. 1996; Brooks 1997; Burgman et al. 2001; Van der Lee et al. 2006).

In this study, we illustrate the application of uncertainty analysis (UA) (Saltelli et al. 2004) to propagate these input uncertainties to the calculated HSI scores. An additional important method useful to evaluate uncertainty is sensitivity analysis (SA), which identifies the most important model parameters (Homma and Saltelli 1996; Saltelli et al. 2004, 2008). Global sensitivity analysis (GSA) refers to a family of SA techniques where the entire parametric space of the model is explored simultaneously for all model inputs. GSA contrasts with more traditionally used local SA measures obtained from “one-parameter-at-a-time” (OAT) methods, that is, they quantify the effect of a single parameter by assuming all others are fixed (Saltelli et al. 2005, 2008). Local sensitivity indices are calculated as derivatives of output in respect to input at single point of input definition (i.e., a nominal point within a plausible range). Therefore, they are only efficient if relationships between all model inputs and outputs are linear and monotonic (Saltelli and Annoni 2010), an assumption often violated in natural systems. Unlike local OAT analysis, GSA can be applied to any model, regardless of model assumptions (although some approaches assume lack of correlation among variables). Furthermore, GSA allows for simultaneous variation of all inputs, thereby providing information not only about the direct (first order) effect of the individual factors over the output, but also about their interaction (higher order) effects (Saltelli et al. 2008). The formal application of combined GSA and UA allows the modeler to estimate model uncertainty, examine model behavior, identify important inputs and interactions between them, select input parameters for model calibration, and prioritize which inputs should be measured or estimated more accurately (Saltelli et al. 2008). The results of GSA can provide valuable guidance in designing further field studies for improving parameter estimates or for validation tests (Van der Lee et al. 2006).

This study demonstrates the application of combined global sensitivity and uncertainty analyses (GSA/UA) for evaluation of habitat suitability models. The presented techniques have been previously applied in other domains, such as safety assessment (Saltelli and Tarantola 2002), chemical (Saltelli et al. 2005), and hydrological modeling (Tang et al. 2007; Pappenberger et al. 2008), and recently in the field of ecological modeling (Lagerwell et al. 2014). Monte Carlo-based UA has been applied to explore propagation of uncertainty of model inputs on outcomes of habitat suitability models (Van der Lee et al. 2006). Johnson and Gillingham (2004) performed a GSA (using the extended Fourier Amplitude Sensitivity Test, FAST) combined with UA to explore the Resource Suitability Index (RSI) model response to uncertainty of habitat rating indexes obtained from expert opinion. Apart from uncertainty of input data and parameters, HSI models are also associated with uncertainty in the functions relating partial HSI with habitat variables, as knowledge of habitat requirements of a species is usually incomplete. Both types of uncertainty propagate through the model and affect output uncertainty. In this study, we further develop approaches presented by Johnson and Gillingham (2004) and Van der Lee et al. (2006) using the method of Sobol for GSA. The advantage of using this method, as compared to other variance-based methods such as Fourier Amplitude Sensitivity Test, FAST (Cukier et al. 1973) or extended FAST (Saltelli et al. 1999), is that it can be applied to factors with a discrete distribution (with levels representing, e.g., various classes or interval data). This useful property allows the introduction of auxiliary factors into the GSA (Lilburne and Tarantola 2009) that can represent model structural uncertainty (Crosetto et al. 2000). We illustrate structural uncertainty in a simple fashion, as uncertainty around HSI functions, thus incorporating within the GSA/UA framework both model input uncertainty and structural uncertainty. The synergistic application of both the uncertainty of the HSI model (from UA) and the sources of uncertainty (from GSA) provides a more complete understanding of model dynamics and limitations.

**Figure 1:**
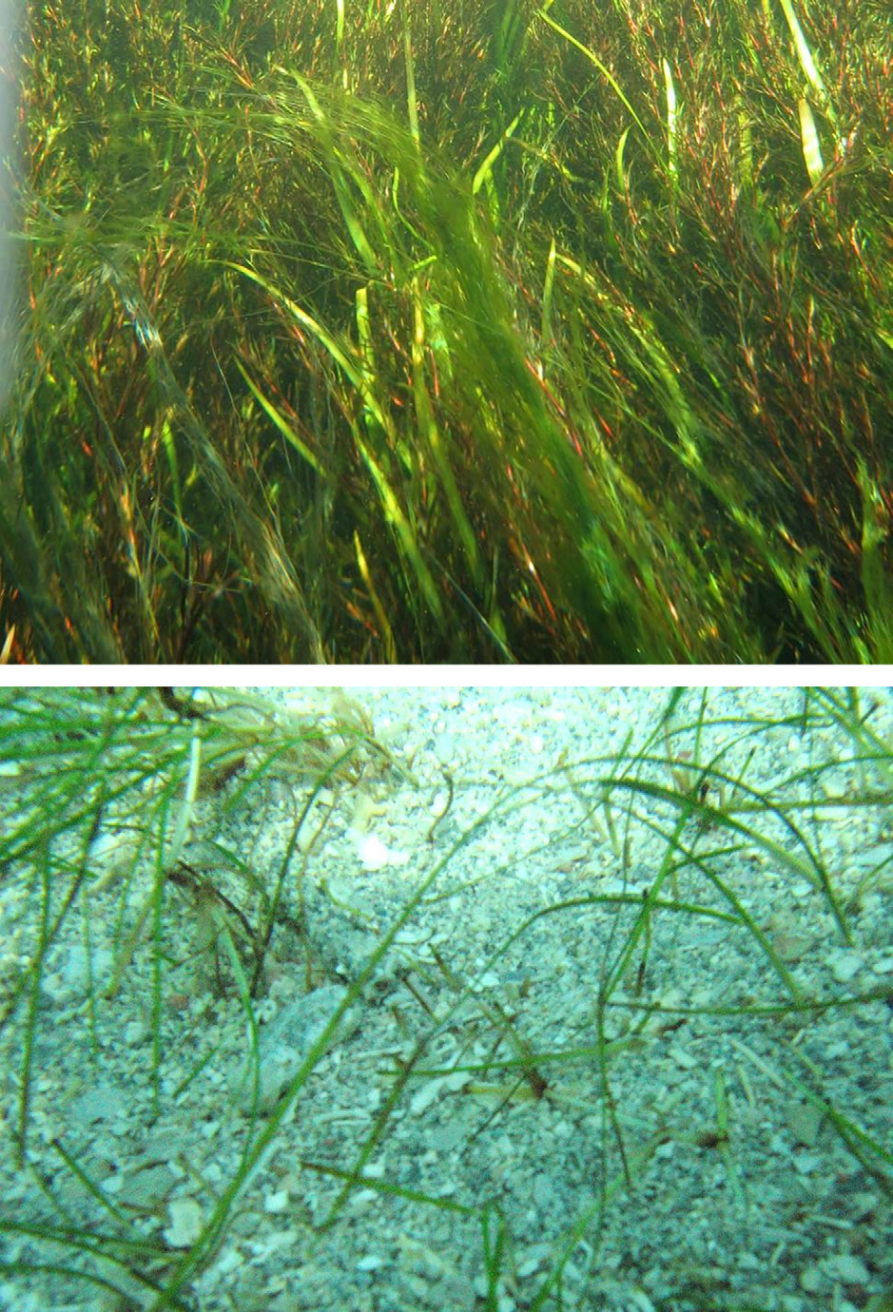
The two SAV species, *Vallisneria americana* (top) and *Halodule wrightii* (bottom).

Our approach also illustrates a method of analyzing large simulated data sets for input to HSI models, thus taking advantage of the increasingly common situation where field data are limiting, but complex climate or hydrologic model output is available to provide simulated data to address landscape scale questions (Jones-Farrand et al. 2011; Nukazawa et al. 2011). Here in an exploratory analysis, we apply the GSA/UA framework to HSI models for two submerged aquatic vegetation (SAV) species: tape grass (*Vallisneria americana*) and shoal grass (*Halodule wrightii*) in a south Florida riverine–estuarine system in Everglades National Park (Fig.1). The two species vary in their habitat requirements, with tape grass being salt-intolerant and shoal grass being salt-tolerant. The application of the GSA/UA to HSI models for these two example species illustrates a method that could be applied to a host of HSI and Spatially Explicit Species Index (SESI) models (Curnutt et al. 2000) which linked to Everglades hydrology restoration and climate change scenarios could provide more informative decision support tools for resource managers. We use data simulated from a hydrodynamic model (water depth, salinity, temperature) as input to our analysis and compare HSI scores and GSA/UA results between current hydrological conditions and future conditions projected from climate and sea level rise scenarios (Swain et al. 2014). We limited our analysis to a subset of possible sources of HSI model uncertainty, emphasizing input data, parameters, and model structure (i.e., shape of HSI functions). We illustrate the results with large-scale maps and graphs for specific sites of interest that show how HSI scores, uncertainty, and sensitivity can vary spatially and temporally. From the results of the two example species, we highlight some issues for resource managers using HSI models for decision support.

## Methods

### Study area

**Figure 2:**
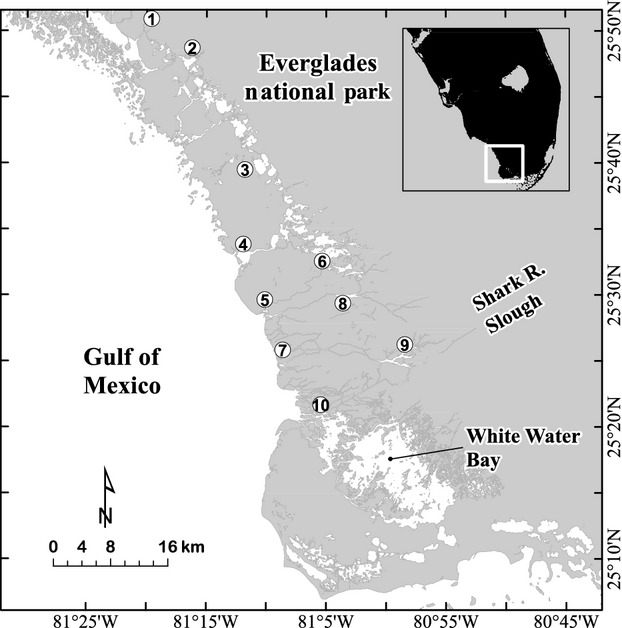
Map of study area. Numbered sites are benchmark locations referred to in subsequent graphs, representing the following locations: 1 = Turner R., 2 = Sunday Bay, 3 = Alligator Bay, 4 = Lostman’s First Bay, 5 = Broad R. Bay, 6 = Rodgers River Bay, 7 = Harney R., 8 = Broad R. Bay, 9 = Tarpon Bay, 10 = Little Shark R.

The study area is the southwestern portion of the Everglades National Park (ENP), Florida, USA (Fig.2). ENP has been the focus of major hydrologic restoration activities, and intensive hydrologic modeling efforts provide massive data sets of hydrologic variables for different restoration and sea level rise scenarios. We relied on a hydrodynamic numerical model (TIME) (Wang et al. 2007) to examine hydrologic variables for each 500 *× *500 m grid cell within the model domain of the study area. The TIME simulations include the period January 1999–December 2047, for current and future conditions with a final sea level rise of 30 cm above current conditions (Swain et al. 2014). Under this scenario, average salinity in the estuaries is projected to increase (unpublished data). Ten sites were chosen for sensitivity analysis, representing estuaries, bays, and major rivers from Turner River in the north (site 1) to lower Shark River (site 10) just north of Whitewater Bay (Fig.2). A major source of freshwater input to the systems occurs via Shark River Slough (Fig.2).

### Construction of the SAV HSI models

**Figure 3:**
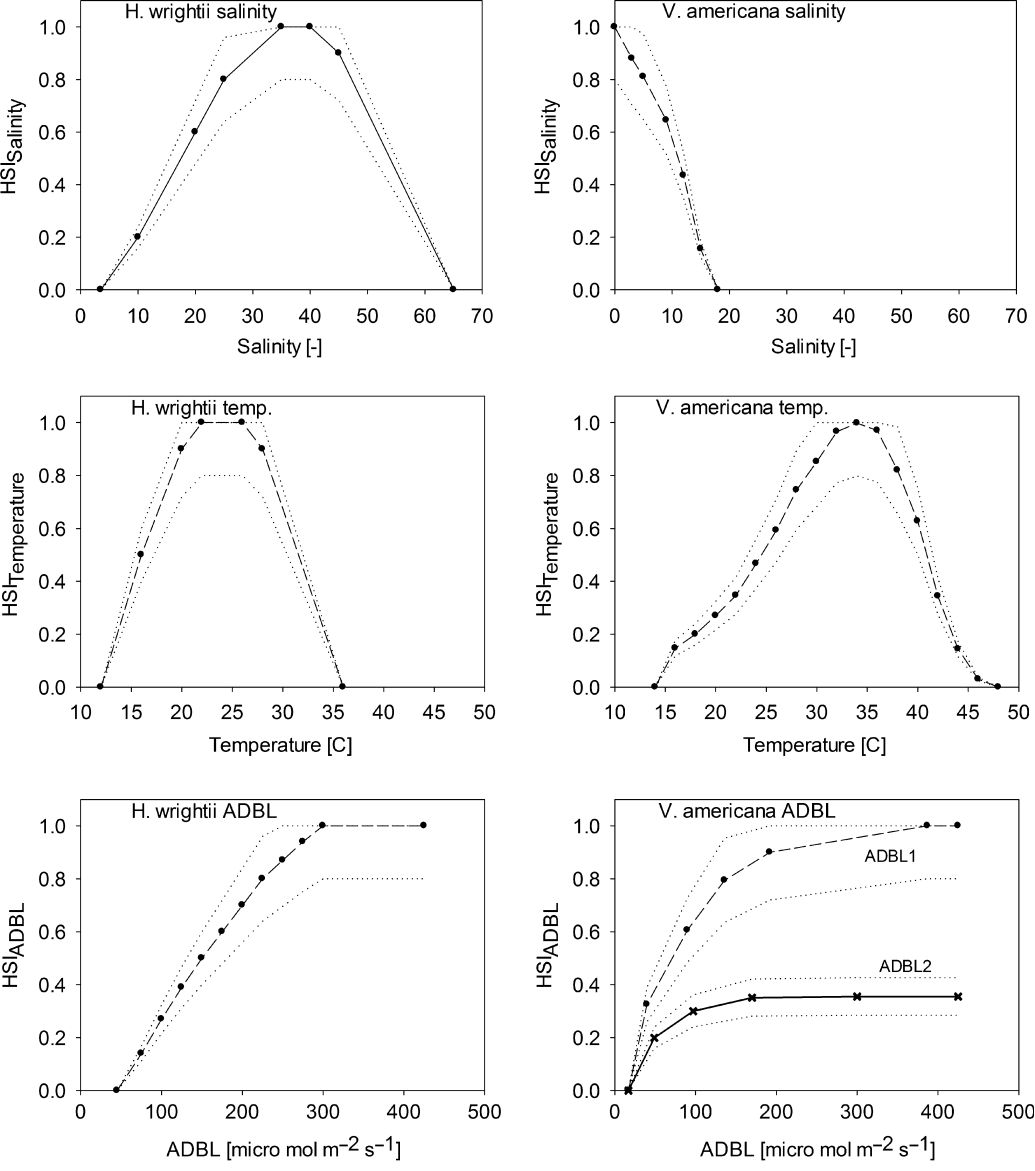
Look-up graphs for salinity, temperature, and light availability used for obtaining partial HSIs with uncertainty ranges (dotted lines) considered for GSA/UA. Graphs on left column are for *H. wrightii*, on right column for *V. americana*. Multiple Average Daily Bottom Light (ADBL) lines for *V. americana* (lower left) are for response under low (ADBL1) or high (ADBL2) salinities. Modified from Mazzotti et al. (2007, 2008).

The SAV species in our analysis have been intensively studied in nearby areas and are known to vary in their habitat requirements for water salinity, temperature, light availability, and other parameters. We relied heavily on Mazzotti et al. (2007, 2008), who used the literature, field, and laboratory data and expert knowledge to develop HSI model functions relating habitat suitability to these parameters (Fig.3) for the two species in the nearby Caloosahatchee River estuarine system of southwest Florida. The HSI model functions were developed by choosing specific life stages of each species with the most limited range of suitable conditions, to capture the highest sensitivities of the organisms to environmental change. Our approach followed other HSI modeling efforts (U.S. Fish and Wildlife Service 1981), by constructing partial HSI models to represent the relationship between each relevant environmental parameter and an associated suitability index (SI). SI values ranged from 0, for fully nonsuitable habitat, to 1, for fully suitable habitat (United States Fish and Wildlife Service 1981). Total HSI was modeled from the partial indices.

The HSI model for the two SAV species used the following five environmental inputs: salinity, temperature, water depth, light attenuation coefficient (*k*), and photosynthetically active radiation (PAR). Input values for salinity, temperature, and water depth were provided by simulations from the TIME model. The hydrology data were provided at 6-h intervals for each parameter for the entire simulation period (January 1997–December 2047), and monthly averages and variances were calculated for each cell for use in the HSI model. The light attenuation coefficient for the water column was initially set at 0.85 m^−1^ as a baseline value for the uncertainty analysis. Average monthly PAR (∼400–700 nm @ *μ*mol m^−2^ sec^−1^) at the water surface was calculated from the daily average PAR as measured at a nearby location in the Estero Bay watershed (lat. 26.35497, long. −81.84453) in nearby Lee County, Florida (Mazzotti et al. 2007, 2008). Light availability was represented by the average daily bottom light (ADBL) values and was calculated using Beers law (Dennison et al. 1993; Onuf 1996) based on water depth, PAR, *k*, and an assumption that 90% of incident light passes through the water surface.

To calculate total HSI for a given location, three partial HSI model indices were calculated for each species using three modeling functions: *salinity function*, *temperature function*, and *ADBL function*, representing lookup functions relating input variables: salinity, temperature, and light availability (ADBL) to habitat suitability (Fig.3). *V. americana* required two functions relating SI to ADBL, as the efficiency of light utilization is conditioned on the salinity as well as water depth (Fig.3 lower right panel). The total HSI index was calculated as the geometric mean of the three equally weighted partial HSI indices, following the approach proposed in Mazzotti et al. (2007, 2008):

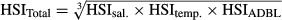
1where HSI_sal._, HSI_temp_, and HSI_ADBL_. are partial SI indices based on salinity, temperature, and light availability, respectively (Fig.3). Partial and total HSI values were calculated on a monthly basis for each grid cell across the study area using monthly means of all inputs for the period January 1997–December 2047. As a result, we obtained maps of total habitat suitability scores for each monthly time step within the simulation period. No spatial correlation was considered, that is, individual cell values were assumed not to be affected by neighboring cells.

### Global sensitivity and uncertainty procedures

**Figure 4:**
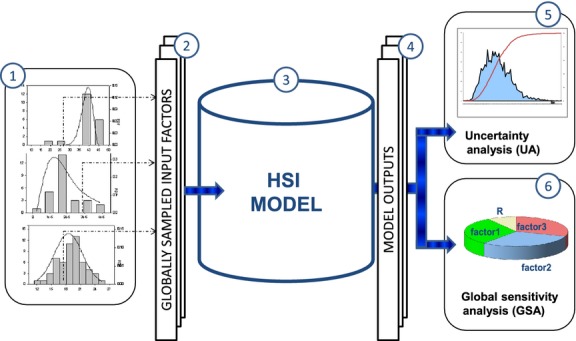
General procedures for global sensitivity and uncertainty analysis (GSA/UA) based on quasi-Monte Carlo (MC) simulations. Steps are labeled as: (1) Define PDF for each input factor, (2) Sample input factors using Sobol method, (3) Perform quasi-MC simulations using HSI model for each input sample, (4) Generate PDFs from model output, (5) Perform uncertainty analysis, (6) Perform sensitivity analysis.

The GSA/UA analysis was performed using the general outline proposed by (Saltelli et al. 2005) as presented in Fig.4 and described in detail below: (1) Probability distribution functions (PDFs) were constructed for each uncertain input factor (model input data, parameters, and functions) based on a uniform distribution; (2) sets of input variables for UA were generated by quasi-Monte Carlo sampling of the multivariate input distribution; (3) HSI model simulations were executed for each input set; (4) uncertainty of model output was assessed for each grid cell of the model domain based on the results from quasi-Monte Carlo simulations; (5) output PDFs and cumulative distribution functions (CDFs) were constructed for select locations; and (6) GSA was performed according to the method of Sobol (1993, 2001).

Specification of model input uncertainty can rely on a variety of information, including empirical measurements, the literature review, expert opinion, physical bounding considerations, etc. (Cacuci et al. 2005; Saltelli et al. 2005). We estimated uncertainty in model inputs and HSI functions using the assumption of a uniform distribution, with ranges of ±20% around inputs’ baseline values and HSI functions (Fig.3). The application of a standardized form and amount of variation (e.g., ±20%) is a common procedure and necessary when estimates of uncertainty from empirical data are unavailable (Fox et al. 2010), as is commonly the case for HSI models. The proportional application of the uniform distribution to the HSI function resulted in smaller SI ranges for lower HSI scores (dotted lines in Fig.3). As SI values cannot fall outside the range from 0 to 1, uncertainty ranges were truncated to 0 and 1 when generated SI values were not contained within this range.

All uncertain input factors (eight for *H. wrightii* and nine for *V. americana*) were sampled by Sobol sequences – quasi random sequences that result in a more homogeneous coverage of sampled parameter input space than Monte Carlo sampling (Sobol 2001). Subsequently, we executed the HSI model for the alternative samples of model inputs and stored corresponding model outputs (e.g., maps of total HSI) for further analysis.

We used the variance-based method of Sobol (1993) that decomposes the total variance of model output between the different uncertain input factors and their interactions, according to the equation (Saltelli et al. 2004):

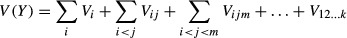
2where *V*(*Y*) is the total variance of the model output *Y*, *V*_*i*_ is the fraction of the output variance explained by the ith model input factor, *V*_*ij*_ is the fraction of the variance due to interactions between factors *i* and *j*, and *k* is the number of inputs.

For a given factor *i*, two sensitivity measures were calculated as follows: the first-order sensitivity index *S*_*i*_, which measures the direct contribution of factor *i* to the total output variance, and the total sensitivity index *S*_Ti_, which contains the sum of all effects involving a given factor (direct effects and effects due to interactions with other factors). The first-order sensitivity index *S*_*i*_ is calculated from the ratio of the output variance explained by the ith model input (*V*_*i*_) to the total output unconditional variance (*V*): 

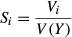
3

Assuming the factors are independent, the total order sensitivity index *S*_Ti_ is calculated as the sum of the first-order index and all higher-order indices of a given parameter. For example, for parameter *X*_*i*_:

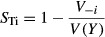
4where *S*_Ti_ is the total order sensitivity, and *V*_−*i*_ is the average variance that results from all parameters, except *X*_*i*_. Sobol indexes are constrained as follows: 0 ≤ *S*_*i*_ ≤ *S*_Ti_ ≤ 1. For a given parameter, *X*_*i*_, interactions with other factors can be isolated by calculating a remainder *S*_Ti_−*S*_*i*_. Factors that have small *S*_*i*_ but large *S*_Ti_ primarily affect model output by interactions. These indexes were calculated by approximate Monte Carlo integrations as described in Saltelli et al. (2008).

The variance-based methods are computationally demanding, as they require a large number of simulations proportional to the number of uncertain factors considered in the GSA. The method of Sobol (1993) requires *M* = (2*k* + 2)*N* model simulations (Lilburne and Tarantola 2009), where *k* is the number of input factors (*k* = 8 and 9 for *H. wrightii* and *V. americana,* respectively), *N* is the number of samples [we used *N* = 512 as suggested by Lilburne & Tarantola (2009)], and M is the total number of iterations. Therefore, a total number of 9216 and 10240 iterations were required for the GSA for HSI models for *H. wrightii* and *V. americana,* respectively. However, no additional computational cost was required to perform UA.

We used the Sobol (1993) approach as implemented in SimLab (ver. 2.2) (a free software for GSA/UA developed by the Joint Research Centre of the European Commission, Saltelli et al. 2004) coupled with R (R Development Core Team 2011) scripts to perform the procedure as outlined in Fig.4. We generated maps of HSI means and standard deviation (SD) for both species for each month of the entire simulation period (based on M maps of HSI values). The maps of standard deviation represent estimated variability around HSI scores as a result of propagated uncertainty from model inputs and due to structural uncertainty associated with the shape of the HSI functions. Other forms of structural uncertainty, such as including additional parameters or using algorithms in addition to the geometric mean were beyond the scope of this study. For the ten benchmark sites, we generated PDF and CDF graphs of the total HSI value for both species for representative dry (March) and wet (June) season months near the beginning (1999, 2002) and end (2045, 2047) of the simulation period, which allowed for visual comparison of temporal changes in suitability associated with projected changes in SLR. We also generated sensitivity graphs for the same time periods at the ten benchmark sites for both species.

## Results

### Spatial variation in HSI mean scores and standard deviation

**Figure 5:**
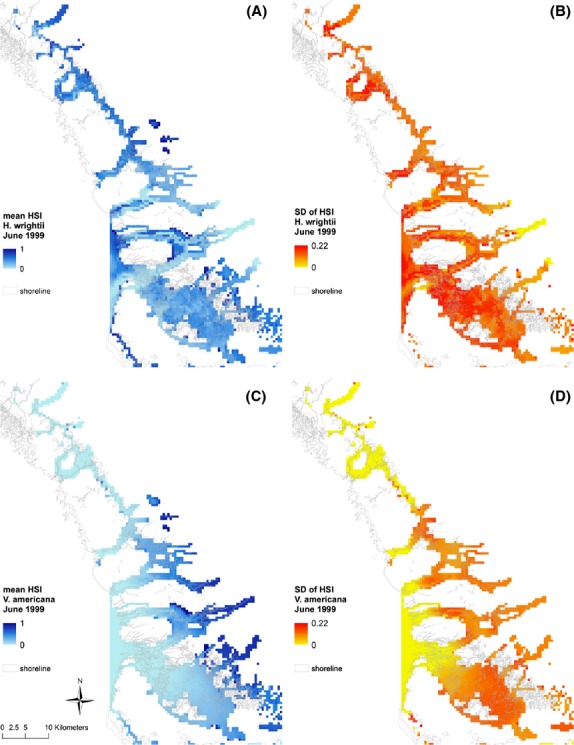
Maps of mean HSI scores (left) and standard deviation (right) for June 1999 obtained from ∼10,000 simulations for *H. wrightii* (A, B) and *V. americana* (C, D).

**Figure 6:**
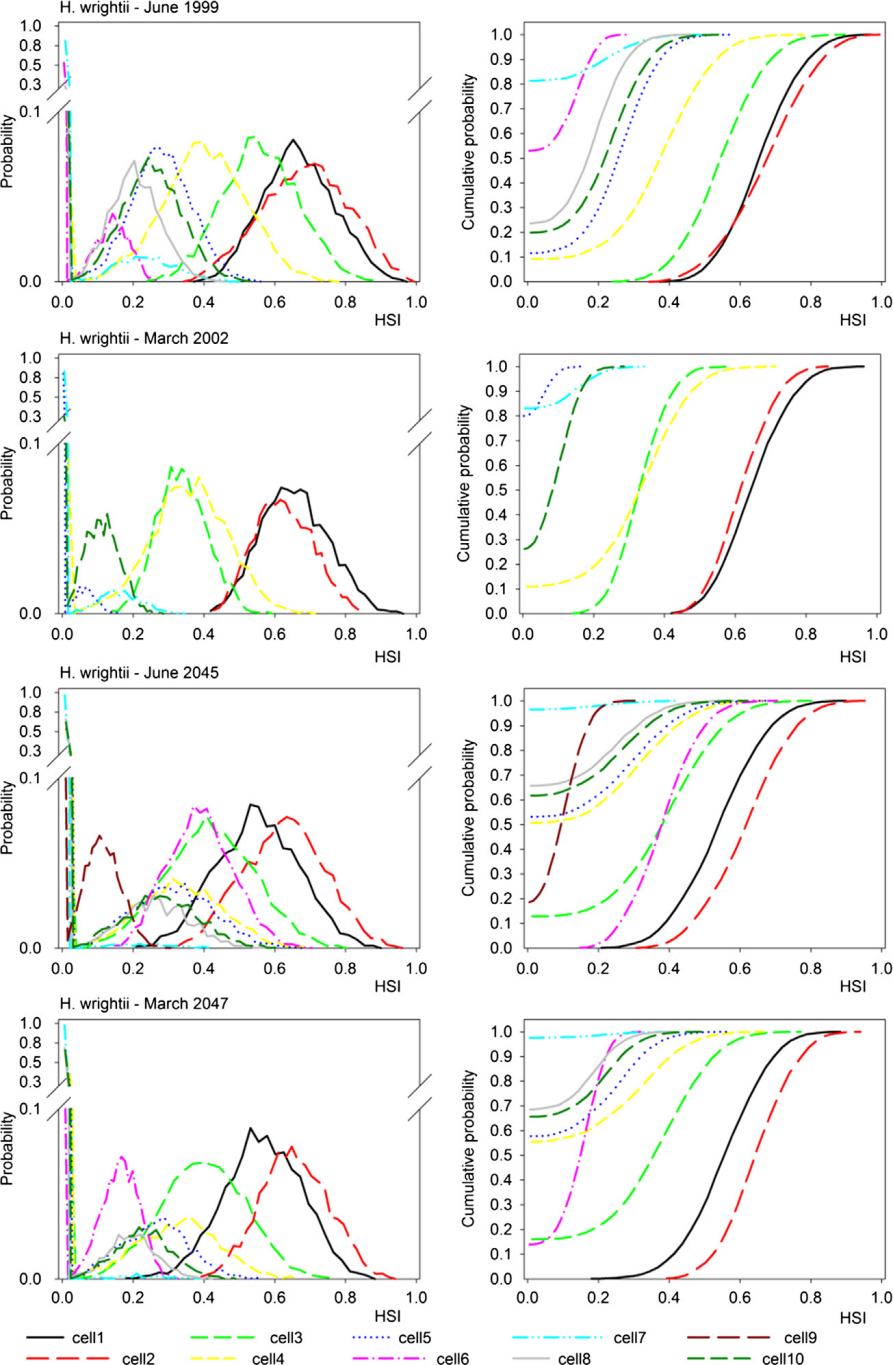
Probability distributions (left) and cumulative distributions (right) for total habitat suitability index (HSI) values for *Halodule wrightii* at selected benchmark sites (i.e., cells) for (top to bottom) June 1999, March 2002, June 2045, March 2047. Some cells have a large number of zero HSI scores that are not visible.

**Figure 7:**
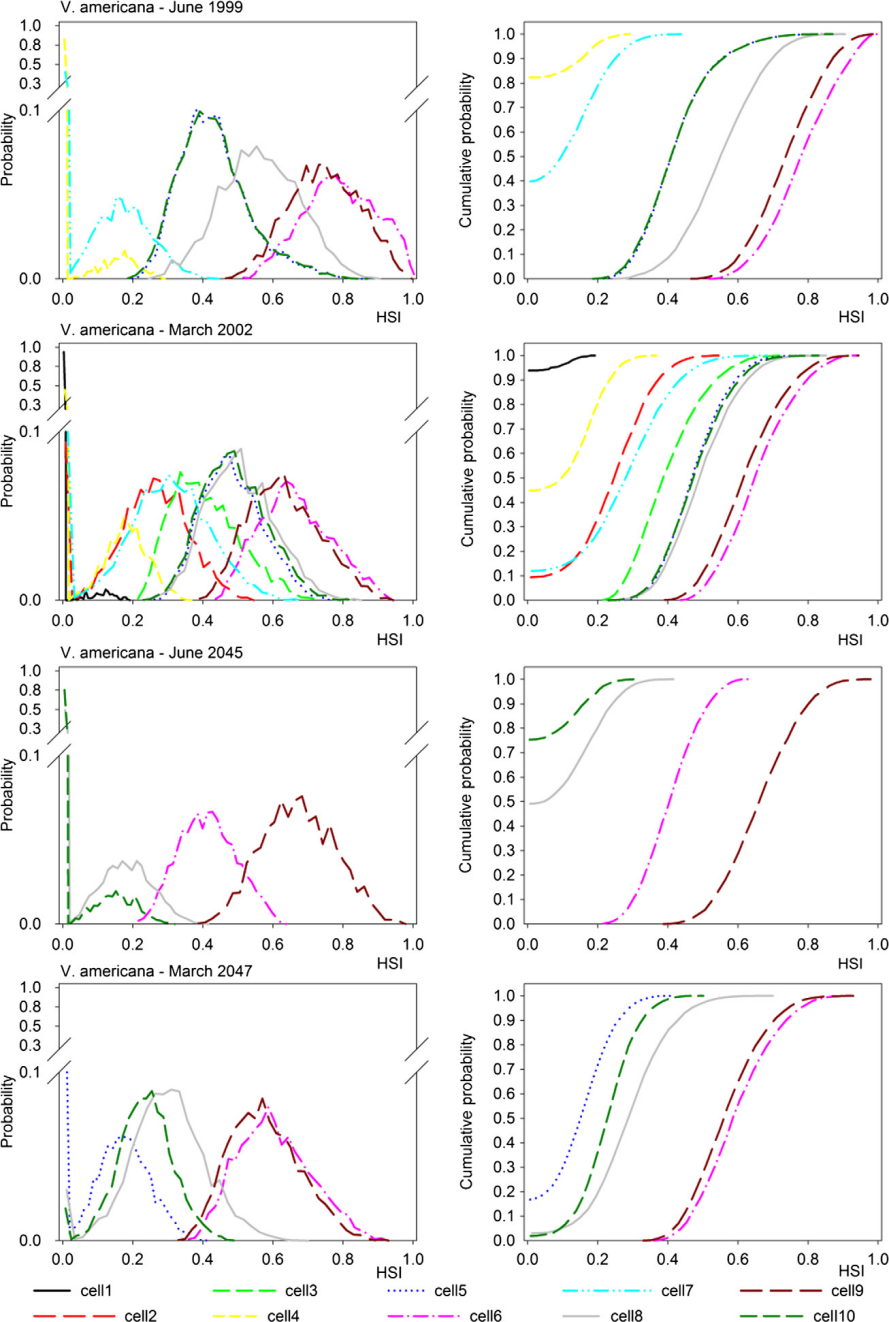
Probability distributions (left) and cumulative distributions (right) for total habitat suitability index (HSI) values for *V. americana* at selected benchmark sites (i.e., cells) for (top to bottom) June 1999, March 2002, June 2045, March 2047.

Example maps of monthly means and standard deviation for the total HSI scores for both SAV species are shown for a typical wet season period (June 1999) in Fig.5. The interpretation of the mapped SDs is relatively straightforward, if mean HSI values are associated with normally distributed output. We found a predominance of approximately normally distributed PDFs at our benchmark sites (see HSI distributions at benchmark sites in Figs.6, 7), suggesting that the mapped uncertainties can be interpreted as SDs or confidence levels on the outputs (Morgan and Henrion 1992). The two species showed different spatial patterns in HSI means, with higher scores for salt-tolerant *H. wrightii* in the bays and Gulf (Fig.5A), and higher scores for salt-intolerant *V. americana* in the inland river systems (Fig.5C). In addition to this differing response for the two species along the inland-Gulf gradient, a northwest–southeast gradient is evident, with higher HSI scores for *H. wrightii* in the northwest, and higher scores for *V. americana* in the southeast. We attribute this pattern to the influence on salinity of the Shark River (Fig.2), which is a major source of freshwater input to the lower half of our study area, creating favorable conditions for *V. americana*.

Spatial variation in uncertainty can be seen in Fig.5B and D, with a general pattern of higher SDs (i.e., uncertainty) in HSI scores for areas with high suitability, and lower SDs in scores for areas with low habitat suitability. Whitewater Bay is a large area that shows a somewhat different pattern, with high uncertainty for both species. The bay receives considerable freshwater influx during typical wet seasons and is subject to tidal flushing which gradually shifts salinity conditions from those favoring *V. Americana* to those favoring *H. wrightii*. The large uncertainties for both species suggest that more intensive monitoring may be necessary in Whitewater Bay.

### HSI distributions at benchmark sites

Examination of PDFs of plausible model outputs provides additional information about forecasted conditions (central value, shape, and spread of the distribution) compared to the more common HSI approach which uses deterministic simulations. Simulated PDFs and CDFs for total HSI scores are compared for the 10 benchmark locations (Fig.2) for typical wet and dry season months (June and March) near the beginning (1999, 2002) and end (2045, 2047) of the hydrology simulations for *H. wrightii* (Fig.6) and *V. americana* (Fig.7). The graphs show which locations are generally more suitable (i.e., have higher scores) and the degree of uncertainty associated with these estimates. The PDFs (left panels of Figs.6 and 7) are approximately normal in distribution for moderate to high-quality sites, whereas poor sites are clustered close to the zero HSI axes with no discernible distribution. The CDFs (right panels of Figs.6 and 7) show more clearly than the PDFs the large number of points clustered at or near the zero HSI axis. The CDFs also show the probability of exceeding a threshold value of interest to management questions and allow easier visual discrimination among sites compared to the PDFs.

For both species, the majority of sites show poor habitat conditions, with sites 1 and 2 scoring highest for *H. wrightii* (Fig.6), and sites 6 and 9 for *V. americana* (Fig.7). These higher scoring sites generally maintained a consistently high ranking at the time slices we investigated, and PDFs usually had little overlap with low scoring sites. There is some evidence for temporal change for *V. Americana*, where sites that are initially marginal to poor in quality deteriorate substantially in the later periods (compare sites 4, 5, & 7 in Fig.7 for June 1999, March 2002 vs. June 2045, March 2047). These marginal to poor sites are near the coast, and the reduced freshwater inflow and sea level rise is expected to further reduce habitat suitability.

### Sensitivity analysis at benchmark sites

**Figure 8:**
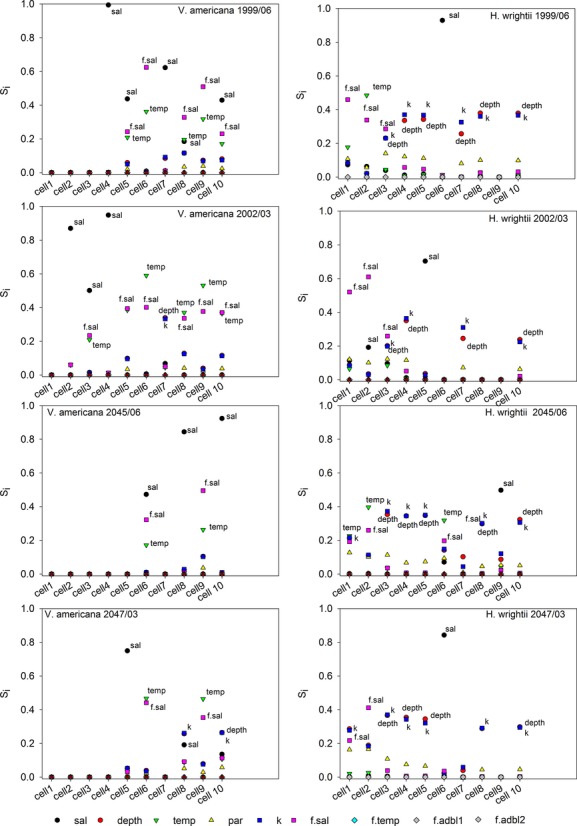
First-order sensitivity indices obtained from ∼10,000 simulations for *V. americana* (left) and *H. wrightii* (right) for selected benchmark cells for June 1999 (top), March 2002, June 2045, March 2047 (bottom); where: sal – salinity, temp – temperature, k – light attenuation coefficient, par – PAR, f.sal, f.temp, f.adbl – salinity, temperature, ADBL functions, respectively.

GSA results comparing the ten benchmark locations for typical wet and dry season months (June and March) near the beginning (1999, 2002) and end (2045, 2047) of the hydrology simulations are presented in Fig.8. The sensitivity indices show the relative contribution of each parameter to the total output variability. The GSA figures depict the first-order effects (*S*_*i*_), defined as a fraction of the total output variance explained by each parameter (vertical axis) for each benchmark cell HSI score (horizontal axis). Higher-order effects were relatively small or negligible (not shown) for sites with relatively high total HSI scores (for all time slices considered). This indicates that all factors contributed to the output uncertainty individually, with little interaction. Several sites with extremely low total HSI scores did show some higher-order interactions, but these values may be the result of numerical error associated with near-zero values.

For a given species–season combination, sites with high scores generally showed sensitivity to different parameters compared to low-quality sites. Among good sites for *V. americana* (sites 6 & 9) *salinity function* and *temperature* scored high for both wet and dry seasons, and remained important from the start to the finish of the simulation (Fig.8 left panels). Among good sites for *H. wrightii* (sites 1 & 2) *salinity function* scored high for both seasons, and *temperature* also scored high for the warmer wet seasons (Fig.8 right panels). Both species showed high sensitivity to salinity, but not at the same sites. *H. wrightii* showed greater sensitivity to more parameters than *V. americana*, most notably *depth* and *k* parameters. This may be associated with the fact that *V. americana* occurs in shallow streams, whereas H. wrightii generally occurs in deeper bays and coastal areas. For both species, the *temperature function* and *ADBL function* scored zero for all sites and time slices. Factors with SI scores at or near zero can be considered unimportant at that site, as they account for little or none of the variation or uncertainty, and are less important parameters from a parameter estimation standpoint. This suggests that the *temperature function* and *ADBL function* can be varied within the assumed uncertainty ranges without much effect on model results.

## Discussion

The strong spatial patterning in the maps of mean HSI scores showed regions with good habitat suitability within the study area. For the two SAV species, there was little overlap in high scoring areas, as would be expected from their differing salinity tolerances. Higher mean HSI scores for *H. wrightii* occurred in the northeastern bays and Gulf, whereas higher mean scores for *V. americana* occurred in the inland river systems in the southeast portion of the study area (Fig.5), which are influenced by freshwater inflow from the Shark River Slough (Fig.2). These patterns provide useful information for researchers and managers, but in the absence of uncertainty information, the mean values alone do not show the expected range of possible values, which if large enough can alter interpretations of habitat quality. For example, the maps of standard deviation showed relatively large uncertainties for both SAV species within most of the areas with high to medium scores. Areas with relatively high scores but large standard deviation may actually have periods of low suitability due to natural high variation in hydrologic conditions (highly variable input parameters), or high sensitivity to a particular uncertain parameter (and its range), or scores that occur on steep-sloped portions of the HSI curves (i.e., associated with model structure) (Van der Lee et al. 2006). This uncertainty should be considered when interpreting the maps of mean scores.

The PDFs and CDFs of HSI scores for the 10 benchmark sites generally showed little or no overlap for sites with good versus poor conditions, but PDFs of intermediate sites often overlapped both good and poor sites, indicating lack of significant differences for these intermediate sites (Schenker and Gentleman 2001). Only a few sites showed consistently higher scores across multiple years and seasons, possibly due to local conditions being relatively stable and favorable. The high scores for *V. americana* at sites downstream from well-developed dendritic creeks near the Shark River Slough (sites 6 and 10), and high scores for *H. wrightii* in the upper bays (sites 1 and 2) receiving little freshwater inflow, are sensible given the differing salinity tolerances of the two species. The lowest scoring sites were consistently low, and from a management perspective may be poor candidates for restoration actions or may not warrant additional monitoring. These uncertainty patterns would not be evident in a typical deterministic HSI model that provides only point estimates of suitability (Van der Lee et al. 2006; Wilhere 2012).

Spatial variation in uncertainty across the entire area showed patterning for both species, with greater uncertainty generally in areas with higher habitat suitability scores, and much lower uncertainty in areas with low scores. This partly reflects our a priori specification that uncertainty is lowest at extreme scores, where conditions are thought to be very poor biologically. While this assumption may be reasonable and has been made by some researchers (e.g., Burgman et al. 2001), other alternatives have been modeled. For example, Van der Lee et al. (2006) developed uncertainty estimates for HSI function curves by expert elicitation for a freshwater SAV species, resulting in highest uncertainties for intermediate HSI scores. Johnson and Gillingham (2004) compared outcomes from assuming different uncertainty distributions (uniform versus triangular) and found that the two distributions lead to different patch-based rankings of habitat quality. The a priori specification of uniform distribution of uncertainty with a fixed percentage around inputs and functions can be considered a major source of uncertainty for this GSA/UA. The analysis is more realistic and defensible when simulated values are drawn from distributions defined by a sample of repeated observation (see Muñoz-Carpena et al. 2007, for a hydrological model example). In principle, if more data were available we could fit more informative distributions. However, considering that HSIs are typically used for applications with no or poor-quality data, the application of uniform or triangular distribution may be the most feasible option. Elicitation of expert opinion provides a complementary or alternative approach (e.g., Johnson and Gillingham 2004; Van der Lee et al. 2006), but would require a formal survey of experts in SAV ecology, which was beyond the scope of this work. Haan et al. (1998) and Helton (1993) indicated that proper assignment of input ranges is more influential on SA than knowledge of the actual PDF and that the simple distributions (such as uniform or triangular) suffice for exploratory SA studies. We chose to apply a uniform distribution (rather than triangular) as it is more conservative (i.e., results in higher uncertainty estimates and it does not require an estimate of central (i.e., most frequent) parameter value. Another simplification in our approach for generating functions is that we truncate partial HSI scores (generated from uniform distribution) so they are confined within a 0–1 range, otherwise the geometric mean could fall outside of this range. This approach could potentially reduce uncertainty for the highest scores (i.e. >0.83). However, the PDFs at the benchmark sites show no evidence of truncation for high scores and seems unlikely to affect the sensitivity ranking, as the model response is controlled mostly by limiting factors (as discussed below).

The results of the GSA for both species suggest that sites with higher scores responded to different parameters compared to poorer scoring sites. At the benchmark sites where scores were high, the GSA indicated that salinity and temperature were the most important variables, suggesting that monitoring and modeling efforts should focus more on these parameters to reduce uncertainty. Light-related parameters were less important, perhaps because better sites were generally in favorable, shallower areas. Our use of the geometric mean assumes a “limiting factor” approach, where a low score for a single habitat parameter can produce a low score for the total HSI, even if all the other parameters have high scores. To obtain relatively high total scores, sites must have high values for all parameters, and consistently high scores across time may be due to more stable environmental conditions compared to poor sites. Further analysis of the local parameter dynamics would be needed to confirm this hypothesis, but these results illustrate the value of GSA to generate hypotheses and a better understanding of the model.

The GSA/UA adds value to the HSI model predictions as it indicates the magnitude and other characteristics (such as spatiotemporal distribution) of uncertainty associated with model results. While a decade ago, analyses were mostly restricted to deterministic estimates provided by models, current good practice is shifting to include uncertainty with associated predictions. Enumeration of uncertainty provides additional information about the robustness of model results, and this knowledge should be incorporated into decision-making. If model uncertainty is large when evaluating model output, it may signal the need to improve the model or model inputs. In general, for models with high temporal variation in input variables, it may be important to look at temporal variation in sensitivity indices (Vezzaro and Mikkelsen 2012). The GSA results contribute to better understanding of focal parameters controlling SAV suitability in the region and therefore understanding of the simulated processes. The GSA results also indicate which variables should be monitored if model uncertainty is to be reduced, thus providing a link to iterative adaptive management.

Our use of GSA/UA was focused on parameter uncertainty and uncertainty associated with HSI functions. We did not consider other sources of uncertainty which could be important. For example, additional variables such as substrate type, nutrients, and fetch can affect SAV (Fourqurean et al. 2003), but these model components were excluded because data were unavailable for the study area. Model equations defining functional relationships are another source of structural uncertainty (McBride and Burgman 2012). We relied on an unweighted geometric mean to combine different parameter scores, but other researchers have used a weighted arithmetic mean (e.g., Ray and Burgman 2006), or a simple minimum score (e.g., Van der Lee et al. 2006). When choosing among alternative modeling configurations, a common approach is to make more “conservative” assumptions (i.e., those that produce the worst case scenario estimate). The geometric mean is highly sensitive to a limiting factor (Johansson and Greening 2000) and may result in underrepresentation of areas with habitats of low, but still nonzero suitability, due to one variable being on the low end of tolerance. The influence of a limiting factor is especially visible for cases when one of the inputs is at a steep slope of the HSI curve, with uncertainty range containing both tolerable and intolerable values (see also Van der Lee et al. 2006).

In our analysis, we made the simplifying assumption that all model inputs and functions are independent. However, correlations may exist, for example, between the light attenuation coefficient and water temperature. Darker water color and suspended solids absorb solar energy, resulting in raised water temperature (Mazzotti et al. 2007 discuss additional correlations, specific to South Florida systems). We assumed factor independence for two reasons: (1) traditional variance-based methods assume that factors are independent and (2) we had inadequate information to construct the correlation structure for dependent inputs. The assumption of independence of model inputs is the main critique of traditional variance-based techniques when applied to environmental models. To address this critique, some methods have emerged recently that are able to consider a correlation between factors (Xu and Gertner 2008; Kucherenko et al. 2012). Mara and Tarantola (2012) proposed a methodology using a de-correlation (i.e., transformation from dependent into independent variables) of correlated inputs prior to calculating indexes with traditional variance-based methods. For studies where sufficient data are available for constructing correlation structure, such techniques would allow independent contributions of inputs to model response to be identified (Xu and Gertner 2008; Mara and Tarantola 2012).

Recognition of the importance of uncertainty and sensitivity analysis in species and habitat models is increasing among researchers, managers, and policy makers. Different modeling methods may require different UA and SA approaches. Species distribution models (SDM) are increasingly being used to evaluate species response to climate change. Software programs such as MAXENT (Elith and Leathwick 2009) can generate estimates of variance and parameter importance automatically, during the course of statistically fitting environmental parameters to species distribution data (Wiens et al. 2009). Some Bayesian analyses also can produce estimates of sensitivity and uncertainty as a by-product of the analysis (Wilhere 2012). The HSI method we used, and similar approaches based on process-based models (Kearney and Porter 2009) or expert elicitation (Johnson and Gillingham 2004), may require explicit effort to produce a sensitivity and uncertainty analysis (Van der Lee et al. 2006).

The choice of which species habitat modeling method to use depends on a variety of factors, including availability of species distribution and environmental input data, and availability of expertise needed to run different types of models and statistical analyses. Where feasible, a multi-modeling approach can be employed, and comparisons of results from different models can provide a measure of model uncertainty as the extent of consensus among the various models (Burgman et al. 2005; Araújo and New 2007; Jones-Farrand et al. 2011). However, multimodeling approaches may be difficult or impossible for many situations where limited data or modeling expertise is available. Distribution data are unavailable for most species (Kearney and Porter 2009), and development of HSI or other simple models that rely on expert knowledge or known habitat relationships may be the only reasonable and practical option (Johnson and Gillingham 2004; Wilhere 2012). In such situations where distribution data are unavailable, it may be especially important to conduct a GSA/UA.

In this analysis, we focused on HSI models for just two species, demonstrating the GSA/UA approach and how results and interpretations of HSI scores can vary due to uncertainty. However, management decisions most frequently now and into the future will be applied to multiple species and communities at varying landscape scales. For example with the restoration of the Florida Everglades, simulation experiments implemented in large-scale hydrodynamic models are providing resource managers with multiple scenarios of changing hydrology conditions under various restoration options (Obeysekera et al. 2011) and climate change and SLR scenarios (Swain et al. 2014). Outputs from these simulations are and will be integrated into previously developed HSI models and SESI models linked to Everglades hydrology. When projecting future habitat suitability for a given scenario a cascade of models is used: results from a regional circulation model input to a regional hydrological model input to the regional habitat suitability model. The uncertainties from all these models propagate through the final HSI model and affect the final uncertainty of the habitat suitability projections. With the GSA/UA techniques presented here, the uncertainty propagated through the models can be evaluated, and the main sources of uncertainty identified. Output from HSI models can provide information to decision-makers on habitat suitability for multiple species under multiple scenarios (Peterson et al. 2003; Weeks et al. 2011). The uncertainty and sensitivity analyses graphics we have shown here can provide a visual means of simultaneously examining uncertainty for multiple species within a community or habitat. Increasingly, data viewers are being developed as decision support tools that can display and compare model outputs from competing management plans (Romanach et al. 2014). Similar graphics from uncertainty and sensitivity analyses would add to the value of these platforms. Failure to consider uncertainty may lead to actions that ignore likely alternative outcomes, possibly resulting in poor decisions or the misdirection of scarce conservation resources (Burgman et al. 2005). Including an uncertainty and sensitivity analysis in modeling efforts as part of the decision-making framework will result in better-informed, more robust decisions.
